# Effect of Milk Protein–Polyphenol Conjugate on the Regulation of GLP-1 Hormone

**DOI:** 10.3390/foods13121935

**Published:** 2024-06-19

**Authors:** Huda Abdulrahim Wazzan, Amanda N. Abraham, Noshin Saiara, Sushil Anand, Harsharn Gill, Ravi Shukla

**Affiliations:** 1 Food and Nutrition, School of Human Science and Design, King Abdulaziz University, Jeddah 21589, Saudi Arabia; hwazzan@kau.edu.sa; 2 Bioscience and Food Technology, School of Science, RMIT University, Bundoora, VIC 3083, Australia; sush.anand@gmail.com; 3 Sir Ian Potter NanoBioSensing Facility, NanoBiotechnology Research Laboratory (NBRL), RMIT University, Melbourne, VIC 3001, Australia; amanda.abraham@rmit.edu.au (A.N.A.); noshinsaiara1993@gmail.com (N.S.); 4 Centre for Advanced Materials & Industrial Chemistry, RMIT University, Melbourne, VIC 3001, Australia

**Keywords:** functional foods, milk, lactoferrin, EGCG, GLP-1, polyphenol, obesity

## Abstract

Modern functional foods are designed to provide health benefits beyond basic nutrition. They are enriched with bioactive ingredients like probiotics, vitamins, minerals, and antioxidants. These foods support overall health, enhance immune function, and help prevent chronic diseases. Milk proteins and tea are known to influence satiety and regulate body weight. Studies have shown that green tea polyphenols, namely, (−)-epigallocatechin gallate (EGCG), and whey proteins, predominantly lactoferrin (LF) from milk, play a role in regulating satiety. This study aims to investigate the effect of conjugating EGCG with apo-lactoferrin (Apo-LF) and assessing these effects on satiety through monitoring glucagon-like peptide-1 (GLP-1) regulation in a human colon (NCI-H716) cell line. Apo-LF-EGCG conjugates were synthesized and characterized in terms of structural and functional properties. The effect on GLP-1 regulation was assessed by real-time quantitative reverse-transcription polymerase chain reaction (qRT-PCR) and enzyme-linked immunosorbent assay (ELISA) to monitor gene and protein expressions, respectively. The results revealed that the protein–polyphenol interaction occurs through the complex formation of hydrogen bonds at the O-H and carbonyl groups of EGCG. The conjugates also showed a significant up-regulation of gene and protein expression levels of GLP-1 while also preventing EGCG from degradation, thereby preserving its antioxidant properties. The Apo-LF-EGCG conjugates increase satiety via increasing GLP-1 secretion in human colon cells while simultaneously retaining the antioxidant properties of EGCG. Therefore, these conjugates show potential for use as dietary supplements to enhance satiety.

## 1. Introduction

Obesity has become an epidemic and a major health problem worldwide. According to the World Health Organization (WHO), the incidence of obesity increased two-fold between 1980 and 2015 [1,2,3]. According to the World Obesity Atlas 2022 data published by the World Obesity Federation, approximately 1 billion people will be obese globally by 2030 [4]. Obesity has been identified as a major risk factor for developing chronic diseases such as metabolic disorders, diabetes, heart disease, and some cancers [5], and it is considered the fifth leading cause of death, resulting in at least 2.8 million deaths annually [1,2,3]. It is also linked to high blood pressure, high cholesterol, especially low-density lipoprotein (LDL) and triglyceride levels, and lowered high-density lipoprotein (HDL) levels [6,7]. Various infectious diseases are also associated with increased body weight. Recently, it was found that obesity is highly interrelated with the most infectious disease, COVID-19, and its associated mortality. COVID-19-related death rates increased ten times in countries where over 50% of the population is overweight or obese [8]. These characteristics of obesity have negative effects on quality of life, work efficiency, and healthcare costs. Thus, there is increasing interest in finding a suitable solution to this global problem.

Over the years, many strategies have been applied to manage and reduce the incidence of obesity through changes in diet and lifestyle and the use of pharmacological agents or surgical interventions. The most common strategy is increasing energy expenditure, which results in weight loss through the modification of dietary habits and a consistent physical activity regimen [3,9]. A complementary approach to achieving weight loss is using weight loss drugs or “Pharmacotherapy”. Drugs such as Orlistat (Xenical) and sibutramine “Meridia” have been approved for weight loss by the Food and Drugs Administration (FDA) [3,10]. However, the usage of these drugs is associated with a range of side effects such as increased blood pressure and heart rate, and they can also cause vitamin A and E deficiencies [3,9]. Bariatric surgery is used as an option that can be performed on the stomach and/or intestine to reduce food intake, which promotes weight loss and reduces health problems such as Type 2 diabetes [11]. However, it is considered a last resort solution as it is an invasive surgical approach, with many adverse side effects and high costs. The modulation of appetite through the regulation of satiety using dietary approaches is now being explored as a safer method to regulate body weight [12,13].

Modern functional foods are designed to provide health benefits beyond basic nutrition. They are enriched with bioactive ingredients like probiotics, vitamins, minerals, and antioxidants. Examples include fortified cereals, probiotic yogurts, and omega-3-enriched eggs. These foods support overall health, enhance immune function, and help prevent chronic diseases. Milk is a high-viscosity beverage, and it is composed of two major protein fractions, viz. casein and whey [14]. These protein fractions have been reported to increase satiety and reduce food consumption by increasing the secretion of satiety hormones such as glucagon-like peptide-1 (GLP-1) and cholecystokinin (CCK) [15,16,17,18,19]. Of the two protein fractions, whey proteins are more efficient at increasing satiety hormones since they contain a higher proportion of branched-chain amino acids and, hence, greatly increase the blood amino acid profile on digestion [20,21]. This, in turn, leads to an increase in the secretion of satiety hormones as amino acids can positively regulate satiety hormones in the gut, where GLP-1 and peptide YY (PYY) are secreted by enteroendocrine L cells [20]. Thus, the GLP-1-secreting NCI-H716 cell line is important to study the behavior of enteroendocrine L cells [22]. They stimulate insulin secretion after meals and once activated, they help to reduce body weight by promoting satiety and delaying gastric emptying [23]. These hormones are secreted rapidly in response to the ingestion of food and eventually promote satiety and help to reduce body weight.

Similarly, green tea beverages rich in phenolic phytochemicals are known to have a significant impact on body weight regulation and energy expenditure [24,25,26,27,28]. Green tea is a rich source of flavonoids, which mainly comprise catechins and their derivatives, including (+)-catechin (C), (−)-epicatechin (EC), (−)-epigallocatechin (EGC), (−)-epicatechin gallate (ECG), and (−)-epigallocatechin gallate (EGCG) [29,30,31], with EGCG accounting for the highest catechin concentration (50 mg in 100 mL) of green tea [32]. Since these polyphenols are extensively degraded when in the gastrointestinal environment, one study assessed the effect of combining green tea polyphenols with dairy products [33], which led to the development and commercialization of numerous beverages such as green tea lattes. The results of the study showed that green tea polyphenols formed complexes with whole milk proteins and significantly improved polyphenol stability [33]. Zeng et al. studied the stability of tea polyphenols at different pH values and temperatures [34]. Atomic force microscopy was performed to investigate the EGCG polyphenol–protein complex, which changes the structure of proteins and was found to be applicable as functional ingredients in foods [35]. Hursel and Westerterp-Plantenga also showed that when a protein is combined with green tea, it could help in the maintenance of body weight following weight loss in moderately obese subjects [36]. Furthermore, a study conducted by Stojadinovic et al. suggested that this protein–phenol interaction could have a positive effect on phenol stability by protecting it during the digestion process [37]. In recent times, studies found that lactoferrin supplementation, a multifunctional glycoprotein in mammalian milk, helps to control weight and obesity in humans [38].

While these studies suggest the positive implications of combining milk proteins with green tea polyphenols in maintaining the stability of polyphenols, there are no reports on the effects of this combination on satiety hormone regulation. Therefore, this study aimed to achieve two primary objectives. Firstly, to explore the conjugation of a whey milk protein, apo-lactoferrin (Apo-LF), with EGCG. Secondly, to assess the impact of a protein–polyphenol conjugate on GLP-1 secretion by human colon cells compared to unbound components. The antioxidant activity of the EGCG in the conjugates was also assessed. This study provides an understanding of the physio-chemical characteristics of the protein–polyphenol conjugate, particularly regarding changes in the protein and polyphenol structures during conjugation while also determining the effects on satiety hormone regulation.

## 2. Materials and Methods

### 2.1. Required Chemicals and Reagents

The Apo-LF sample from bovine whey was gifted by Tatura Milk Industries Ltd. (a subsidiary of Bega Cheese, Tatura, Australia). EGCG, sodium phosphate dibasic (Na_2_HPO_4_), sodium phosphate monobasic dehydrate (NaH_2_PO_4_), bovine serum albumin (BSA), 2,2′-Azino-bis (3-ethylbenzothiazoline-6-sulfonic acid, potassium persulfate, (±)-6-Hydroxy-2,5,7,8-tetramethylchromane-2-carboxylic acid (ABTS), and dialysis tubing cellulose membrane (12 kDa cut-off) were purchased from Sigma-Aldrich (Castle Hill, NSW, Australia). A phosphate buffer solution (PBS, pH 7.0) was prepared by mixing 30.5 mL of 0.2 M Na_2_HPO_4_ with 19.5 mL of 0.2 M NaH_2_PO_4_ made up to 100 mL. A dialysis membrane was used after sensitizing by boiling in distilled water. RPMI 1640 medium, DMEM medium, fetal bovine serum (FBS), 3-(4,5-dimethylthiazol-2-yl)-2,5-diphenyltetrazolium bromide (MTT), Trizol reagent, and Geltrex™ hESC-Qualified ready-to-use basement membrane matrix was purchased from Life Technologies (Mulgrave, VIC, Australia). High-Capacity cDNA Reverse Transcription Kits, TaqMan fast universal PCR master mix, FAM-MGB probe/primer set for GLP-1 (Hs01031536_m1) and β-actin (Hs02742610_g1), donkey anti-mouse polyclonal antibody tagged with Alexa Fluor^®^ 488 added (secondary antibody for ELISA), Hoechst 33342, gold anti-fade mounting medium, and RIPA lysis and extraction buffer were purchased from Life Technologies (Mulgrave, VIC, Australia). Deionized Milli-Q water was used for all experiments.

Human NCI-H716 cells were obtained from the American Type Culture Collection (Manassas, VA, USA). Cells were grown in suspension in RPMI 1640 supplemented with 10% FBS, 2 mM L-glutamine, 100 IU/mL penicillin, and 100 μg/mL streptomycin. Cell adhesion was initiated by growing cells in dishes coated with Matrix DMEM, 10% FBS, 2 mM L-glutamine, 100 IU/mL penicillin, and 100 μg/mL streptomycin.

### 2.2. Apo-LF-EGCG Conjugate Synthesis

The conjugates were prepared according to Liu et al. (2016) with slight modifications [39]. Briefly, a stock solution of 20 µM Apo-LF in PBS was prepared and stirred for 2 h at room temperature (RT). Also, an EGCG (1 M) stock solution was prepared in PBS. Both the Apo-LF and EGCG solutions were mixed to achieve a final concentration of EGCG ranging from 15.63 to 500 µM, with a constant Apo-LF concentration of 10 µM, continuously stirring at RT for 24 h with free exposure to air. The samples were then dialyzed against water for 24 h, frozen at −80 °C, and then lyophilized using an OPERON freeze dryer (Gimpo, Republic of Korea) operating at −55 °C to form solid samples. These samples were used for all further experiments.

### 2.3. Characterization of the Apo-LF-EGCG Conjugates

Fourier transform infrared (FTIR) spectroscopy was measured using a GladiATR Single Reflection spectrometer (PIKE Technologies, Inc., Fitchburg, WI, USA) in the 400−4000 cm^−1^ range with a resolution of 4 cm^−1^. Twenty scans were performed for each measurement, and the conjugate spectra were normalized evenly. Circular dichroism (CD) spectra were recorded using a JASCO J-1500 CD spectrometer (JASCO, Tsukuba, Japan), under constant nitrogen flush, from 190 to 300 nm. A quartz cylindrical cell with a 0.1 cm path length was used, and spectra were obtained at a speed of 100 nm/min. Each spectrum is presented as an average of three consecutive measurements. Changes in the secondary structure of Apo-LF were analyzed using an online program to determine the secondary structure (DichroWeb, http://dichroweb.cryst.bbk.ac.uk (accessed on 24 September 2023). Zeta (ζ) potential measurements of Apo-LF-EGCG conjugates were determined using a Malvern Zetasizer Nano ZS (Malvern Panalytical Ltd. Malvern, UK). Samples were loaded into a capillary cell, and the data collected were an average of 100 sequential readings, at a temperature of 25 °C. Scanning electron microscopy (SEM) was used to study the microstructure of lactoferrin (LF) protein before and after its conjugates with EGCG. Freeze-dried samples were gently mounted on SEM stubs covered with double-sided carbon tape and sputter-coated with a fine layer of gold. The microstructure of LF samples was examined using a Philips XL30 digital scanning electron microscope (FEI Technologies Inc., Hillsboro, OR, USA) operated at 30 kV accelerating voltage.

### 2.4. HPLC-MS Analysis

To quantify the EGCG concentration in the conjugates, a conventional HPLC-MS system (Agilent Technologies 1200 Series, 6410 Triple Quad LC/MS) equipped with a Zorbax Eclipse Plus C18 analytical column (2.1 × 50 mm) packed with 1.8 μm particles was used. The mobile phases consisted of 0.1% formic acid in water (A) and 0.1% formic acid in methanol (B). The linear gradient elution system was 75% of A and 25% of B, 2 min at a flow rate of 0.3 mL/min, and the temperature was maintained at 30 °C. EGCG standards ranging from 3.90 to 500 µM in PBS were used to obtain a standard curve. The EGCG content in the Apo-LF-EGCG conjugates was determined by quantifying the EGCG content in the supernatants after centrifuging the conjugates at 10,000 rpm for 10 min. All measurements were run in triplicates. The percentage of absorbance at 734 nm was calculated and plotted against the concentration of catechins (EGCG) using the following formula:

EGCG concentration in supernatant (%) = (AC − AS)/AC × 100

where AC is the absorbance of the control and AS is the absorbance of the samples.

### 2.5. Fluorescence Spectroscopy Analysis

LF proteins have intrinsic fluorescence, which comes mainly from the tryptophan (Trp) and tyrosine (Tyr) amino acids in the protein [40]. Since any changes in the microenvironment of these amino acids will be reflected as changes in their fluorescence spectra, fluorescence spectroscopy of the tryptophan molecules was used to investigate the interaction between Apo-LF and EGCG during conjugation. The mechanism of interaction and the thermodynamic parameters can be determined by monitoring the fluorescence spectra under different temperature conditions [41]. Fluorescence measurements were carried out using a Horiba FluoroMax-4 fluorescence spectrophotometer (HORIBA Instruments Inc., Edison, NJ, USA), and spectra were recorded with the excitation wavelength at 285 nm with emission from 295 to 500 nm since these are the conditions under which Tyr produces fluorescence [40]. Both excitation and emission slit widths were set at 1 nm. In addition, temperature-dependent studies were conducted using the internal temperature control on the instrument, and fluorescence spectra were collected from 20 to 70 °C at intervals of 10 °C.

The fluorescence quenching data were analyzed by fitting the Stern–Volmer equation (Equation (1)).

Fo/F = 1 + K_sv [Q](1)

where Fo and F refer to the fluorescence intensities in the absence and presence of the quencher and [Q] is the concentration of the quencher. Ksv is the Stern–Volmer constant [42,43,44].

The binding constant Kq and the number of binding sites n can be calculated according to a double-logarithmic equation (Equation (2)).

log (Fo − F)/F = LogK_q-n log[Q](2)

The intercept of the double logarithmic Stern−Volmer plot provides the binding constant (Kq), and the slope gives the number of binding sites (n).

For non-linear regression, the thermodynamic forces responsible for the binding of EGCG to Apo-LF were analyzed by a polynomial fit [45]. The dependence of lnK on 1/T is represented by the following equation:

InK = α0 + α1/T + α2/T2(3)

where K is the binding constant at the corresponding temperature (T) in Kelvin.

The thermodynamic parameters can be calculated using the following equations:

ΔH° = −R(α1 + 2 α2/T)(4)

ΔS° = R(α0 − α2/T2)(5)

ΔG° = ΔH° − TΔS°(6)

where R is the gas constant (8.314 J/K mol), ΔH° and ΔS° correspond to the changes in enthalpy and entropy, respectively, and ΔG° corresponds to the Gibbs free energy.

### 2.6. Assessment of Antioxidant Activity

The antioxidant activity of EGCG was determined using ABTS^•+^ decoloring assay as described in Re et al. (1999) [46]. A stock solution of 7 mM of ABTS^•+^ was prepared, and ABTS^•+^ free radicals were produced by reacting ABTS^•+^ stock solution with 2.45 mM of potassium persulfate. The solution was stored in the dark at RT for 12–16 h before use. ABTS^•+^ working solution was obtained by diluting it with PBS to an absorbance of 0.70 (±0.02) at 734 nm. Trolox standards were prepared fresh each time in ethanol at a final concentration between 0 and 15 µM, and appropriate solvent blanks were incorporated where necessary. The reaction mixture of the standard and conjugates was obtained by mixing 200 µL of ABTS^•+^ working solution with 20 µL of each sample or standard. All measurements were run in triplicates. The percentage of absorbance at 734 nm was calculated and plotted against the concentration of antioxidants of Trolox using the following formula:

ABTS^•+^ scavenging activity (%) = (AC − AS)/AC × 100 (7)

where AC is the absorbance of the control and AS is the absorbance of the samples.

### 2.7. MTT Assay

Cells were seeded at a concentration of 1 × 10^4^ cells/well in 96-well culture plates coated with Matrix gel. After 24 h, the cells were treated with the test agents (Apo-LF, EGCG, or Apo-LF-EGCG conjugates) at increasing concentrations in complete media. Then, 0.5 mg/mL MTT solution was added to the cells in serum-free media and incubated in the dark for 4 h at 37 °C. The formazan crystals generated were dissolved in 100 µL of acidified isopropanol, and the absorbance was measured at 570 nm using a SpectraMax Paradigm Multimode Microplate Reader (Molecular Devices, LLC. San Jose, CA, USA). The viability of the cells in each treatment was calculated using Equation (8). An average of three independent experiments was used, and each experiment contained three replicates.

Cell viability (%) = AS/AC × 100(8)

where AC is the absorbance of the control and AS is the absorbance of the samples.

### 2.8. qRT-PCR

A total of 1.5 × 10^6^ cells were seeded in 12-well culture plates coated with Matrigel (Geltrex). The cells were treated with test agents at different concentrations in media containing 0.2% (*w*/*v*) BSA for 18 h. Glucose was used as a positive control. Trizol reagent was used for RNA extraction, and the method was followed as per the manufacturer’s instructions. cDNA conversion was performed using a High-Capacity cDNA Reverse Transcription Kit. qRT-PCR was performed on a 7500 Fast Real-Time PCR system (Applied Biosystems, Melbourne, Australia), using β-actin as the housekeeping gene. Data are expressed as fold change in the target gene determined using the 2^−ΔΔCt^ method [47].

### 2.9. ELISA

For the protein expression study, the ELISA protocol of Chen and Reimer was adopted with minor modifications [17]. In brief, cells were seeded at 2 × 10^6^ cells/well in 12-well culture plates coated with Matrigel. The cells were treated for 3 h with different concentrations of test reagents in media containing 0.2% (*w*/*v*) BSA. The media were collected and saved for measurement of the extracellular release of GLP-1. The cells were washed with cold PBS twice, and 500 µL of RIPA buffer was added to the cells with the addition of 50 mg/L of phenylmethylsulfonyl fluoride and 34 mg/L of diprotin A. The cells were incubated for 15 min on ice. Cells were then collected and centrifuged at 14,000× *g* for 15 min to pellet the cell debris. GLP-1 protein was measured as described by the supplier of the ELISA reagents kit purchased from BD Bioscience (Singapore). Anti-GLP1 primary antibody and donkey anti-mouse polyclonal secondary antibody tagged with HRP were purchased from Abcam, Melbourne, Australia.

### 2.10. Statistical Analysis

Statistical analyses were performed using Graph Pad Prism 7. A *p*-value ≤ 0.05 is considered significant. The data in the figures are presented as means ± standard errors of the mean.

## 3. Results and Discussion

The Apo-LF-EGCG conjugates were synthesized and analyzed. The external morphological changes in the conjugated protein were analyzed through SEM (Figure S1). In the absence of EGCG, Apo-LF had a smoother surface morphology (Figure S1a) compared with the conjugates. A similar morphology of bovine LF was previously reported [48]. Upon binding with EGCG, the surface of Apo-LF becomes uneven and affects its microstructure. The morphology of the Apo-LF-EGCG conjugate presented an irregular spherical structure, and its surface was relatively rough. HPLC-MS analysis performed on supernatants of the conjugates suggests that less than 1% of EGCG was found in the supernatants (Figure S2, Table S1).

The molecular interaction between EGCG and Apo-LF was further confirmed by surface charge analysis. We found that the ζ-potential of Apo-LF was affected in the presence of EGCG. Apo-LF is a basic protein with a high isoelectric point (pH 8.5) and has a positive charge at physiological pH [49,50]. The ζ-potential of Apo-LF was found to be 8.86 mV in this study. On the other hand, the ζ-potential of EGCG was found to be −13.23 mV because of the lower isoelectric point (pH 4.28) of EGCG. The ζ-potential of Apo-LF was drastically decreased from the pristine condition in the presence of EGCG and decreased with increasing EGCG concentrations (Table S2). An earlier study showed that the addition of green tea polyphenols to proteins does not affect the ζ-potential of proteins [51]. However, we and others found that the conjugation of polyphenols changes the surface charge of lactoferrin [52]. These preliminary observations suggest that the changes in the physiochemical properties of Apo-LF occur during the binding process, which are further investigated below.

### 3.1. Interaction between EGCG and Apo-LF

**Figure 1:**
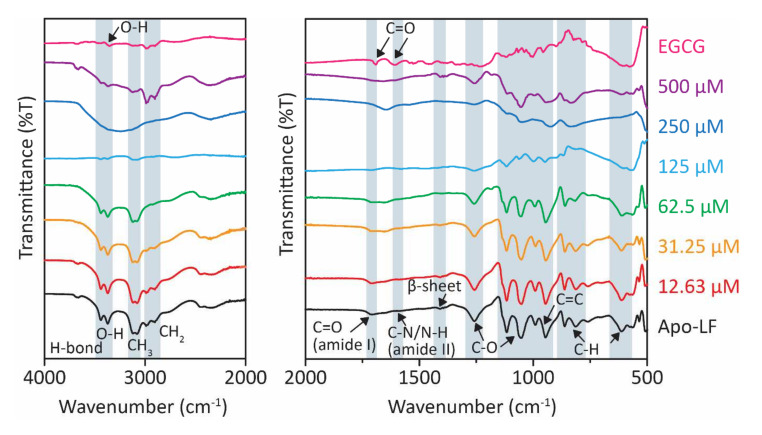
FTIR spectra of Apo-LF-EGCG conjugates show the effect of increasing concentrations of EGCG (15.62–500 µM) on Apo-LF at pH 7.

FTIR, a non-destructive technique, was performed to assess changes in the protein and EGCG structures after conjugation. The peaks at 3550, 3476, and 3355 cm^−1^ refer to the phenolic groups of EGCG (Figure 1). In the conjugate, at lower concentrations of EGCG (15.16, 30.25, 62.50 µM), these peaks undergo a shift to lower wavelength, while at higher concentrations (125, 250, and 500 µM), these peaks are replaced by a broad feature at ca. 3300 cm^−1^. This suggests the involvement of the O-H groups of EGCG in complex formation with Apo-LF. Moreover, two nearby peaks at 1691 and 1616 cm^−1^ represent the carbonyl stretching of EGCG [44]. These observations are consistent with previous findings showing marked differences between native EGCG and Apo-LF-EGCG conjugates [35,41]. At the same time, a noticeable change in the chemical compositions of Apo-LF was observed in the Apo-LF-EGCG complex. The peaks between 3435 and 3368 cm^−1^ are associated with O-H and N-H vibrations involved in hydrogen bonds [53]. Changes in the Apo-LF structure were also investigated. The doublets at 3112/3085 and 2978/2900 cm^−1^ indicate symmetric and asymmetric stretching of CH_3_ and CH_2_, respectively [41,44]. The amide I peak at 1705 cm^−1^ refers to the C=O stretching of the Apo-LF secondary structure. Also, the peak at 1580 cm^−1^ corresponds to N-H bending and C-N stretching of the amide II bond [52]. There is a shift to a higher wavelength in this region, in the case of the conjugates. This suggests that hydrophobic interactions are involved in the formation of Apo-LF-EGCG conjugates [54]. Apo-LF also has a peak at 1410 cm^−1^, which confirms the presence of β-sheet in the structure [53]. Peaks between 1260 and 1048 cm^−1^ are associated with the C-O bond stretching of the carboxyl group of the protein in (R-CH_3_) [44]. These peaks shifted to lower wavelengths in the conjugates. Apo-LF also has a band at 945 and 815 cm^−1^ that corresponds to C=C and C-H stretching vibrations, respectively [44,52]. While it remained unchanged in the presence of lower EGCG concentrations (15.16, 30.25, 62.50 µM) in the conjugates, it shifted to higher wavelengths with higher EGCG concentrations (125, 250, and 500 µM). These changes indicate modifications to the secondary structure of Apo-LF, which are further investigated by CD spectroscopy below.

### 3.2. Changes in the Secondary Structure of Apo-LF

The protein conformational analysis based on CD data indicated that the addition of EGCG led to a change in the secondary structure of Apo-LF, as shown in Table 1. The CD spectra are shown in Supplementary Information Figure S2. Although pristine LF is composed of 33–34% α-helices and 17–18% β-strands [55,56], in this study, we found that Apo-LF had 30.7% α-helices and 20.23% β-sheets (Table 1). Following treatment with different concentrations of EGCG, there was a concentration-dependent decrease in the α-helices content of Apo-LF and an increase in β-sheets. Apo-LF changes the secondary structure when conjugated with EGCG up to 250 µM. A gradual decrease in the % of α-helix content in the complex suggests possible covalent interactions with EGCG that lead to changes in the secondary structure of the Apo-LF protein. The protein assumes a slightly loose conformation due to the increase in unordered coil fractions. These results were consistent with previous findings that also found that in the presence of EGCG, α-helices decreased with increasing β-sheets of LF protein [35,57]. Interestingly, the complex made of the highest concentration of EGCG consistently did not follow the concentration-dependent trend in α-helix content (Figure S2b). This may be possible due to the shielding effect of EGCG that shows a CD pattern similar to pure EGCG (Figure S2a). The higher EGCG concentration seems to over-saturate all the binding sites available on the protein. Taken together, Apo-LF changes its secondary structure when conjugated with EGCG as it assumes a slightly relaxed conformation due to the increase in unordered coil fractions [58].

### 3.3. Antioxidant Capacities of Conjugates

**Figure 2:**
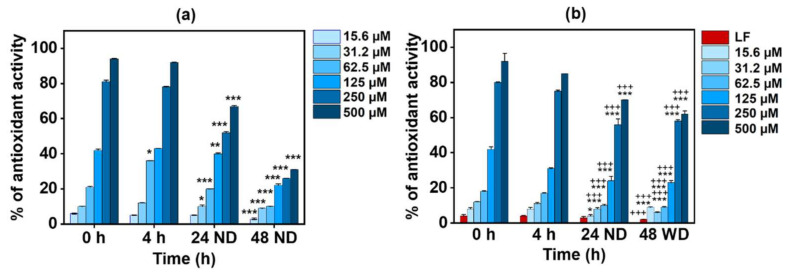
Time-dependent antioxidant activity of (**a**) EGCG alone and (**b**) the Apo-LF-EGCG conjugate at 0 h, 4 h, and 24 h with no dialysis (ND) and 48 h with dialysis (WD) using ABTS^•+^ decolorization assay. * *p* < 0.05, ** *p* < 0.001, and *** *p* < 0.0001 in comparison with 0 h. +++ *p* < 0.0001 in comparison with the corresponding concentration of free EGCG. Here, LF refers to lactoferrin.

There are some conflicting observations regarding the effect of milk on the bio-efficacy of polyphenols in tea. Some studies found that the addition of milk proteins to polyphenols decreased their antioxidant capacity [59,60,61], while other reports found no such effect [54,62]. Hence, in this study, the antioxidant potential of EGCG in the conjugates was quantified. The results in Figure 2 depict the antioxidant activities of pristine EGCG and the conjugates at 0 h, 4 h, and 24 h before dialysis (24 ND) and after a further 24 h of dialysis (48 WD). The results indicate that pristine EGCG showed a concentration-dependent increase in antioxidant activity; however, there was no significant difference in activity between 0 h and 4 h (Figure 2a). There was a significant decrease in the activity of EGCG after 24 h (24 ND), particularly evident by the 20% decrease in activity observed at the higher concentrations (250 µM and 500 µM). This might be due to the significant degradation of EGCG after 24 h [63,64]. After a further 24 h incubation (48 ND), some free EGCG was either lost or further degraded, leading to a further decrease (~70% decrease) in the antioxidant activity in comparison with 0 h.

Figure 2b shows that the Apo-LF-EGCG conjugate has the same antioxidant profile up to 24 h (24 ND) as free EGCG (Figure 2a). This suggests that the antioxidant properties of the conjugates are from EGCG alone since pristine Apo-LF showed no significant antioxidant potential. Studies have shown that LF has antioxidant properties only at concentrations above 1 M [56,65], whereas the concentration of LF used in this study was 10 µM. The results also indicate that the EGCG antioxidant properties are not hampered during the conjugation process (Figure 2b). Interestingly, after dialysis (48 WD), the conjugates showed the same level of antioxidant activity as before dialysis (24 ND), and this was significantly higher (*p* < 0.0001) than the antioxidant activity of the free EGCG after dialysis. This suggests that EGCG is firmly bound to Apo-LF and, hence, protected from degradation and/or loss during the dialysis process. Similar preservation of green tea polyphenol antioxidant activity by milk proteins was previously reported [35,57,66,67]. However, none of these studies showed this behavior with single milk protein conjugates.

### 3.4. Mechanism of Interaction in the Conjugates

The aromatic residues of Tyr and Trp contribute to hydrophobic interactions that stabilize the main structure of protein interiors because they have relatively large polar surface areas [44,68]. Tryptophan residues are often found fully or partially buried in the hydrophobic region of the protein at the interface between two protein domains or subdomains [42,44]. Hence, measuring changes in the inherent tryptophan fluorescence emission spectra will provide information for understanding structural changes during the conjugation of Apo-LF when conjugated with EGCG [44]. It can also provide insight into the binding mechanism and thermodynamic parameters involved in the conjugation process [69].

**Figure 3:**
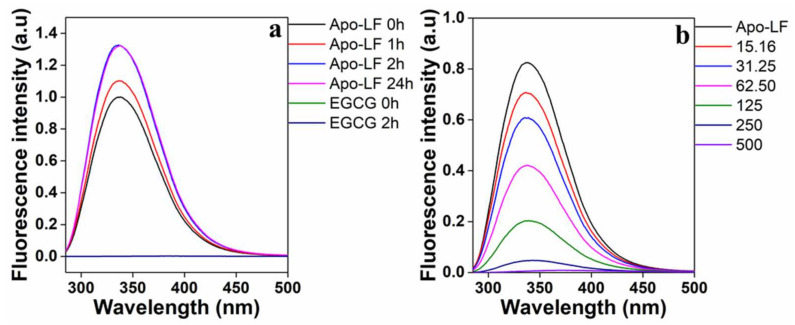
(**a**) Time-dependent fluorescence emission spectra of Apo-LF to optimize solubilization of Apo-LF and (**b**) concentration-dependent effect of EGCG on Apo-LF-EGCG during conjugation. The Apo-LF concentration is 10 µM.

The fluorescence spectra of the tryptophan residues in Apo-LF in the presence of increasing EGCG concentrations are shown in Figure 3. To optimize the length of time required to solubilize Apo-LF in PBS, fluorescence spectra were collected at various time points from 0 to 24 h. The fluorescence reached maximum intensity after 2 h and remained constant up to 24 h (Figure 3a); hence, Apo-LF was dissolved for 2 h before it was used in the conjugation process. It should be noted that pristine EGCG at the highest concentration used for conjugation did not show any fluorescence with the excitation wavelength (285 nm) used in this study. This indicates that the observed spectra are solely from Apo-LF. Figure 3b shows a decrease in fluorescence with increasing EGCG concentrations, which implies that there is concentration-dependent binding between Apo-LF and EGCG. If the Typ emission peak undergoes a blue shift, it indicates a change in the protein structure toward a more hydrophobic environment, while a redshift indicates a change toward a more hydrophilic environment, possibly resulting from the unfolding of the protein [70]. In this case, there is a minor red shift in the Apo-LF spectra as the EGCG concentration increases, which is clearly visible at higher concentrations. This revealed that there was an immediate change in the environment of the tryptophan residues and a hydrophilic change in the secondary structure of Apo-LF in the presence of EGCG [41,71].

**Figure 4:**
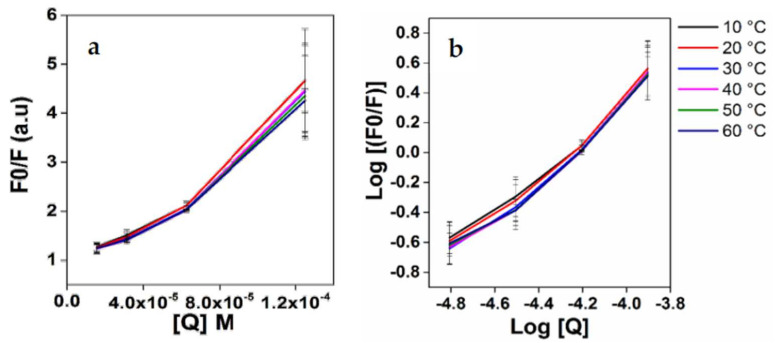
(**a**) Stern–Volmer of F_o_/F vs. EGCG and (**b**) double logarithmic regression curves of log [(Fo-F)/F] versus log[Q].

The above quenching can occur through two different mechanisms, namely, static quenching, i.e., ground state conjugate formation, or dynamic quenching, i.e., collisional quenching. This can be determined from Stern−Volmer plots, from which the Stern–Volmer constants (*K_sv_*) were determined using Equations (1) and (2). Table 2 summarizes the results, and Figure 4a shows the Stern−Volmer plots. Only concentrations that fell within the linear range of the Stern−Volmer regression curve were used to generate the *Ksv* data. From Table 2, it is evident that the *K_sv_* values for Apo-LF-EGCG show a decrease with an increase in temperature. This indicates that the quenching mechanism is through static binding, where the EGCG forms a ground state conjugate with Apo-LF [69]. The fluorescence lifetime of Trp is 10^8^ s (τ_0_), and k_q_ was calculated using the formula k_sv_/τ_0_ [68]. Since the k_q_ values we obtained are in the order of 10^13^ M^−1^ s^−1^ (Table 2), which is above 10^10^ M^−1^ s^−1^, this further confirms that the quenching resulted from static more than dynamic quenching [69].

The double logarithmic regression curves of log [(Fo-F)/F] versus log [Q] were plotted for the Apo-LF-EGCG conjugates (Figure 4b), the intercept of which gives *K*, the binding constant. Using this binding constant, the thermodynamic forces responsible for the binding of LF protein to EGCG were determined using Equation (3). The binding constants (K) for the Apo-LF-EGCG conjugates indicate the binding capacity increases as temperature rises. The K values for the complexes showed an increase followed by a decrease in temperature; these results revealed that the conjugation of phenolic compounds with proteins might have changed the net charge of protein molecules, which in turn influenced the hydrophilic, hydrophobic, and surface properties of the protein. As listed in Table 2, Apo-LF-EGCG conjugates exhibit positive ΔH° and positive ΔS° values. This is indicative of hydrophobic forces [69]. The negative ΔG° also indicates the spontaneity of the binding of EGCG to Apo-LP during conjugation [69]. Furthermore, a similar study using Apo-LF and EGCG estimated that the binding of the polyphenols of EGCG to Apo-LF seems to be more hydrophobic than hydrophilic [44].

### 3.5. Effect of Conjugates on Cell Viability

**Figure 5:**
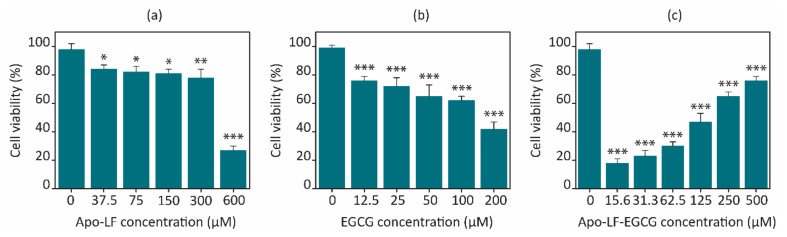
Concentration-dependent effect of (**a**) pristine Apo-LF, (**b**) pristine EGCG, and (**c**) Apo-LF-EGCG conjugates on cell viability of the NCI-H716 cell line after 24 h. Results are the average of three independent experiments, and the error bars represent the standard deviation. * *p* < 0.05, ** *p* < 0.001, and *** *p* < 0.0001 when compared with the corresponding untreated cells.

The effect of pristine Apo-LF, pristine EGCG, and the Apo-LF-EGCG conjugates on the proliferative of NCI-H716 cells was investigated through an MTT assay. Pristine Apo-LF (Figure 5a) showed no significant cell death up to 300 µM. This is to be expected since LF is proven to act as an in vitro growth factor activator [55]. Pristine EGCG (Figure 5b) showed a concentration-dependent increase in cell death, which was expected since EGCG has been shown to exhibit toxicity to various cell lines at similar concentrations [72].

The freeze-dried conjugates were used to treat the cells at concentrations of 0.5 mg/mL in media for 24 h. Interestingly, the Apo-LF-EGCG conjugates showed a concentration-dependent increase in cell death, whereas the conjugates containing less EGCG showed greater cell death than the ones with a higher EGCG content (Figure 5c). A total of 15.63 µM EGCG in the conjugate showed ~20% cell viability, while 500 µM EGCG in the conjugate showed ~75% cell viability. Pristine EGCG, on the other hand, showed 42.36% cell viability at the highest concentration tested (200 µM). The Apo-LF concentration in the conjugates was kept constant at 10 µM, which could not have contributed to the cell death observed with the conjugates since pristine Apo-LF exhibited ~80% cell viability with treatment as high as 300 µM.

**Figure 6:**
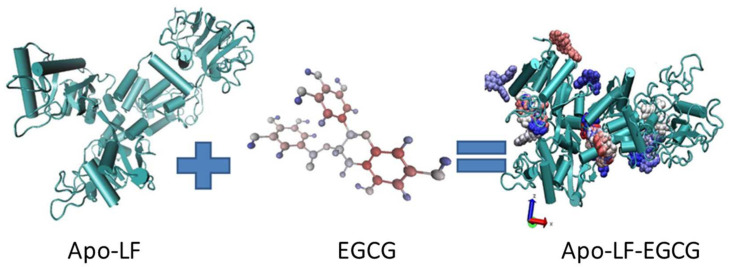
Schematic diagram of the Apo-LF conjugate with EGCG.

This in vitro behavior with the conjugates can be attributed to the concentration-dependent interaction observed between EGCG and Apo-LF. Since the molecule of EGCG is much smaller than Apo-LF, it appears that EGCG might get embedded/inserted into the LF molecule at high concentrations, while it remains surrounding the LF molecule surface at low concentrations. The FTIR, CD, and fluorescence spectra above support this hypothesis since the interaction between EGCG and Apo-LF at lower concentrations caused fewer changes in the secondary structure of Apo-LF. An online simulation software (SwissDock) also showed that EGCG can be embedded inside the milk protein, as shown in Figure 6. The antioxidant properties also suggest that since EGCG is protected from degradation, its activity on the cells is maintained for the entire 24 h of treatment in the conjugates, while free EGCG undergoes degradation and hence has less effect on cell viability at similar concentrations.

### 3.6. Effect of the Conjugate on GLP-1

**Figure 7:**
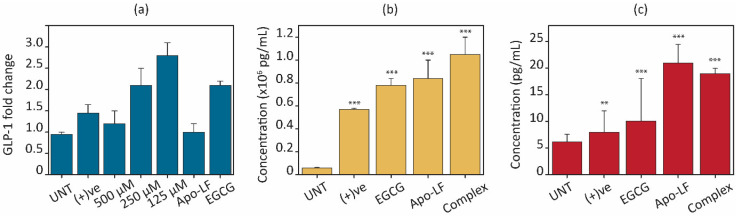
Effects of Apo-LF (10 µM), EGCG (100 µM), and Apo-LF-EGCG conjugates on (**a**) gene expression of GLP-1 hormone after 18 h. (**b**) Intracellular GLP-1 and (**c**) released GLP-1 into the media after 2 h treatment. ** *p* < 0.001 and *** *p*< 0.0001 when compared with corresponding to untreated (UNT) cells.

Several cellular models of animal and human origin have been used to study the regulation of satiety hormone secretion, such as mouse intestine cell lines (STC-1), human colorectal cells (NCI-H716), and murine endocrine cells (GLUTag) [73]. Those modules have provided useful information regarding the signaling pathways that regulate proglucagon gene expression, for example, GLP-1 secretion [74]. GLP-1 is an incretin hormone that is released by enteroendocrine L cells in the gastrointestinal tract (GI). Since NCI-H716 cells have been extensively validated as models to investigate GLP-1 secretion [17], these cells were chosen to study GLP-1 expression. Moreover, NCI-H716 cells release GLP-1 not only in response to nutrient stimulation including fatty acids, amino acids, protein hydrolysates, artificial sweeteners, and bitter compounds but also in response to other hormones such as insulin, leptin, and selected neurotransmitters [75,76]. Hence, NCI-H716 cells were chosen to study the effects of milk proteins on GLP-1. Gene expression was assessed through qRT-PCR on NCI-H716 cells after an 18 h treatment with pristine Apo-LF, pristine EGCG, and the Apo-LF-EGCG conjugates, using 100 µM of glucose as a positive control (Figure 7). The glucose control showed a 1.5-fold increase, similar to that reported by others, indicating the cells were functioning normally [58]. Pristine Apo-LF showed no significant change in the expression of the GLP-1 at 10 µM of the protein. On the other hand, pristine EGCG (100 µM) showed a 1.9-fold increase, indicating that EGCG is capable of increasing GLP-1 gene expression (Figure 7a). There are no known reports of the effect of EGCG on GLP-1 on NCI-H716 cells; however, a similar increase in gene expression was reported in human intestinal (CaCo-2) cells [58].

The Apo-LF-EGCG conjugates with 125 µM of EGCG show the highest effect on enhancing the expression of GLP-1 hormone (2.9-fold increase) compared with 1.8- and 1.35-fold increases for 250 µM and 500 µM of EGCG, respectively. This indicates that since EGCG degradation is prevented when in the conjugate, it might have a better effect on GLP-1 gene regulation, and/or the effect of Apo-LF on GLP-1 is enhanced by the presence of the EGCG in the conjugate.

The secretion of intracellular and extracellular GLP-1 protein contents after 3 h treatment with the pristine components and Apo-LF-EGCG conjugates were assessed by ELISA, and the results are displayed in Figure 7b,c. When GLP-1 concentrations in cell lysate were determined after treatment with free EGCG and Apo-LF, both showed a significant increase (21.58 times with Apo-LF and 18.91 times with EGCG) in intracellular GLP-1 levels in comparison with the untreated cells (UNT) (Figure 7b). The positive control showed a 2.65-times higher increase than the untreated cells. Regarding the conjugate, there was a significant (~27 times) increase in GLP-1 levels compared with the untreated cells. Compared with free Apo-LF and EGCG, the conjugate showed 1.25 times and 1.3 times greater increases, respectively, suggesting that the conjugate works better than the individual components at increasing GLP-1 protein levels.

Apart from intracellular protein content, the extracellular release of GLP-1 into the surrounding cell media was also assessed. This is an important parameter to consider when observing hormone regulation since evidence suggests that cells can store a certain quantity of protein in their cytoplasm. This is released in a “burst” when receptors are activated. This is followed by an increase in gene expression to replenish the cytoplasmic stores of the hormone [73]. Most studies only consider the extracellular release of satiety hormones, which only provides information on the “burst” release and does not consider the gene or intracellular sustained up-regulation of these hormones. Therefore, these studies provide information on short-term satiety regulation and do not consider long-term sustained increases in satiety regulation.

The results from the extracellular release of GLP-1 (Figure 7c) showed that free Apo-LF and EGCG both increase GLP-1 release in comparison with the untreated cells. This trend is similar to that observed for intracellular GLP-1 content, where Apo-LF showed a higher effect than EGCG. Interestingly, while the release of GLP-1 by the Apo-LF-EGCG conjugate was greater than in untreated cells, there was no significant difference when compared to free Apo-LF. These results suggest that while the Apo-LF component of the conjugate might be responsible for the burst release and increase in protein content in the cells, the EGCG in the conjugate could allow for a long-term sustained increase in GLP-1 through regulation of GLP-1 gene expression.

## 4. Conclusions

The present study thoroughly examined the conjugation behavior of Apo-LF-EGCG conjugates, with varying EGCG concentrations (15.63 to 500 µM) and a constant Apo-LF concentration (10 µM), using a range of experimental approaches. Significant structural changes in Apo-LF post-reaction with polyphenols were confirmed by FTIR, CD, and fluorescence spectroscopy, demonstrating that protein–polyphenol conjugation effectively enhances the functional properties, such as antioxidant activity, of phenols. In vitro analysis further highlighted the potential of milk proteins and polyphenols in regulating appetite and controlling energy balance through the secretion of anorexigenic gut hormones. Future studies should check the effect of protein–polyphenol complexes and other bioactive compounds on the expression of GLP-1 hormone in the small intestine. These findings underscore the promise of functional foods in promoting health and preventing disease by leveraging the synergistic effects of bioactive compounds.

## Figures and Tables

**Table 1:** The secondary structure of Apo-LF and Apo-LF-EGCG conjugates.

Sample	Apo-LF (10 µM)	Apo-LF-EGCG Conjugates (µM)					
		15.63	31.25	62.50	125	250	500
*α*-helix	30.7%	31.33%	30.45%	29.29%	23.28%	20.53%	22.63%
*β*-strand	20.23%	18.07%	19.06%	20.34%	26.77%	30.11%	27.61%

**Table 2:** Stern–Volmer constants and thermodynamic parameters of the Apo-LF-EGCG conjugates.

Temperature (°C)	*Kq* (×10^12^)	*Ksv* (×10^4^ M^−1^)	K (×10^2^ M^−1^)	ΔG° (kJmol^−1^)	ΔH° (kJmol^−1^)	ΔS° (Jmol^−1^K^−1^ × 10^13^)
10	3.16	3.16	22.08	−20.62	4.56	3.76
20	3.18	3.18	29.14	−21.51		
30	3.01	3.01	35.92	−22.40		
40	3.03	3.03	34.64	−23.29		
50	2.92	2.92	25.86	−24.18		
60	2.82	2.82	20.71	−25.07		
70	2.89	2.89	17.32	−25.96		

## Data Availability

The original contributions presented in the study are included in the article, further inquiries can be directed to the corresponding authors.

## Supplementary Materials

The following supporting information can be downloaded at: https://www.mdpi.com/article/10.3390/foods13121935/s1, Figure S1: SEM micrographs of (a) freeze-dried Apo-LF, (b) Apo-LF conjugate with EGCG. The surface of Apo-LF became after being treated with EGCG. Scale bar = 2 µm; Figure S2: Standard curve of EGCG; Figure S3: (a) Far-UV CD spectra of Apo-LF-EGCG conjugate at 20 °C. (b) The characteristics ellipticity points indicating the α helix in the Apo-LF-EGCG conjugates are plotted against EGCG concentrations with constant Apo-LF quantity; Table S1: Percentage of EGCG found in the supernatant of Apo-LF-EGCG conjugates; Table S2: The ζ-potential of the conjugate, EGCG and Apo-LF.
