# Supplementary material for: Effect of Milk Protein–Polyphenol Conjugate on the Regulation of GLP-1 Hormone
Source: Foods. 2024 Jun 19;13(12):1935. doi: 10.3390/foods13121935 (PMC11202982; doi:10.3390/foods13121935)
Supplement: Supplementary file 1 [file foods-13-01935-s001.zip › foods-3053297-supplementary.pdf]

Supplementary information for

Effect of milk protein-polyphenol conjugate on the regulation of GLP-1 hormone

Huda Abdulrahim Wazzan [1,2], Amanda N. Abraham [3], Noshin Saiara [3], Sushil Anand [2], Harsharn Gill [2*] and Ravi Shukla [2,3,4*]

Harsharn Gill,[2] and Ravi Shukla[2,3*]

[1] Food and Nutrition, School of Human Science and Design, King Abdulaziz University, Jeddah 21589, Saudi Arabia
[2] Bioscience and Food Technology, School of Science, RMIT University, Bundoora, VIC 3083, Australia
[3] Sir Ian Potter NanoBioSensing Facility, NanoBiotechnology Research Lab (NBRL), RMIT University, Melbourne, VIC 3001, Australia
[4] Centre for Advanced Materials & Industrial Chemistry, RMIT University, Melbourne, VIC 3001, Australia
* Correspondence: ravi.shukla@rmit.edu.au; harsharn.gill@rmit.edu.au

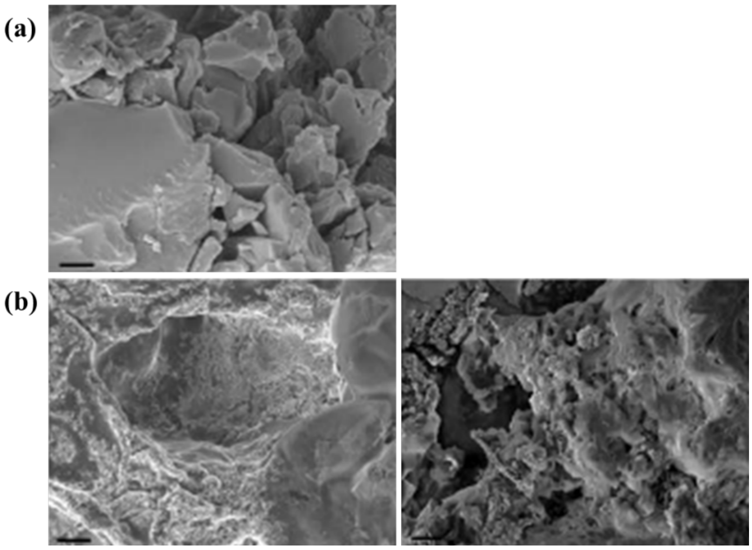

Figure S1. SEM micrographs of (a) freeze-dried Apo-LF, (b) Apo-LF conjugate with EGCG. The surface of Apo-LF became after being treated with EGCG. Scale bar = 2 µm.

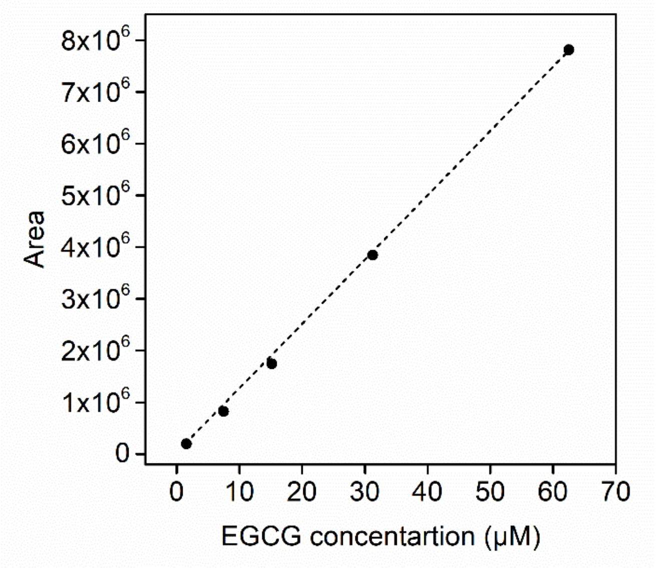

Figure S2. Standard curve of EGCG.

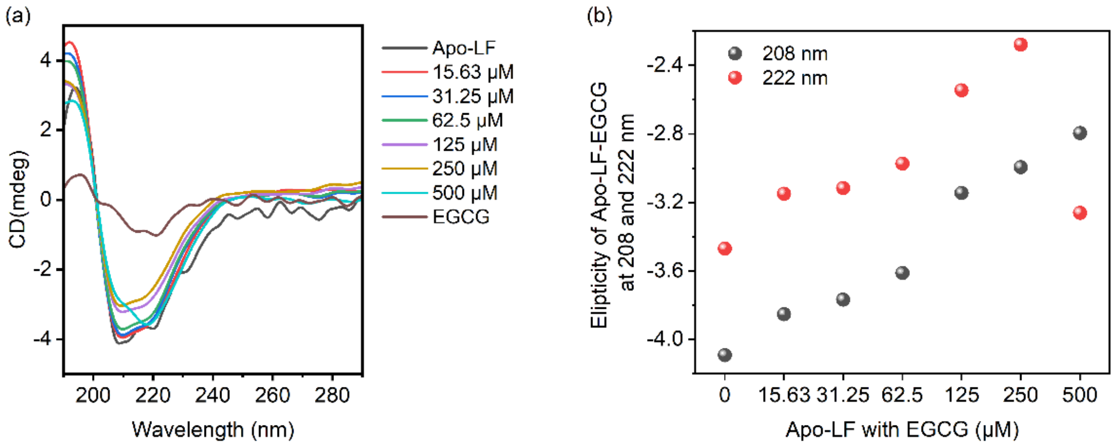

Figure S3. (a) Far-UV CD spectra of Apo-LF-EGCG conjugate at 20 °C. (b) The characteristics ellipticity points indicating the α helix in the Apo-LF-EGCG conjugates are plotted against EGCG concentrations with constant Apo-LF quantity.

Table S1. Percentage of EGCG found in the supernatant of Apo-LF-EGCG conjugates.

EGCG in conjugates(μM)	% EGCG (in the supernatant)
15.63	0.07
31.25	0.04
62.5	0.10
125	0.10
250	0.41
500	0.96

Table S2. The ζ-potential of the conjugate, EGCG and Apo-LF.

Samples	Zeta potential (mV) ± SD
Apo-LF (10 uM)	8.86 ± 0.5
EGCG (500 uM)	-13.23 ± 0.6

RMIT Classification: Trusted

Apo-LF-EGCG (15.162 uM)	-5.33 ± 0.1
Apo-LF-EGCG (32.25 uM)	-6.29 ± 0.43
Apo-LF-EGCG (62.5 uM)	-6.03 ± 0.3
Apo-LF-EGCG (125 uM)	-6.33 ± 0.33
Apo-LF-EGCG (250 uM)	-6.29 ± 0.2
Apo-LF-EGCG (500 uM)	-7.26 ± 0.33
